# A FORGOTTEN STATUS: GOSSYPIBOMA

**DOI:** 10.1590/0102-672020190004e1571

**Published:** 2021-05-14

**Authors:** Burhan Hakan KANAT, Nizamettin KUTLUER, Mehmet Buğra BOZAN, Nurullah AKSOY, Tülin ÖZTÜRK

**Affiliations:** 1 University of Health Sciences, Elazığ City Hospital, Department of General Surgery, Elazig/Turkey; 2 Medical Faculty of Kahramanmaras Sutcu Imam University, Department of General Surgery, Kahramanmaras/Turkey; 3 Siverek State Hospital, Department of General Surgery, Şanlıurfa/Turkey; 4 University of Health Sciences, Elazığ City Hospital, Department of Radiology, Elazig/Turkey

**Keywords:** Gossybipoma, Foreign body, Acute abdomen, Gossibipoma, Corpo estranho, Abdome agudo

## INTRODUCTION

Gossypiboma is used to describe the forgotten cotton/gauze piece in the body after the surgical procedure. It is formed by the combination of the words “gossypium”, a Latin word for cotton, and the word “boma” in Swahili, which means the place of hiding^1^. Although many different materials have been reported in the literature, cotton materials are among the most forgotten objects. Though there is no consensus, the incidence is given as 0.01-0.001%. Gossypiboma, which is more common after abdominal and pelvic surgery, has also been reported after thoracic surgery, spinal, orthopedic, and breast surgery^2^ ^,^ ^3^.

This article aimed to present five gossypiboma cases.

## CASE REPORTS

The patients who were operated with the pre-diagnosis of gossypiboma between February-2012 and October-2018 were retrospectively analyzed. The data were obtained from personal and computer records. Necessary permissions were obtained from the hospital administration and informed consent form was obtained from all patients. Patients who lacked sufficient data were excluded. Gender, initial surgical diagnosis, time passed until diagnosis of gossypiboma and symptoms were evaluated.

**FIGURE 1:**
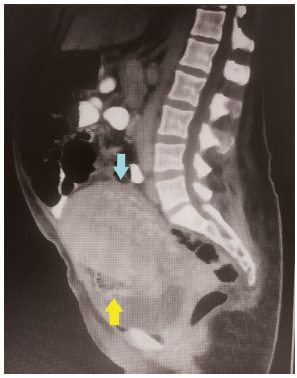
Coronal computerized tomography view of the patient (blue arrow=uterus; yellow arrow=gossybipoma)

**FIGURE 2:**
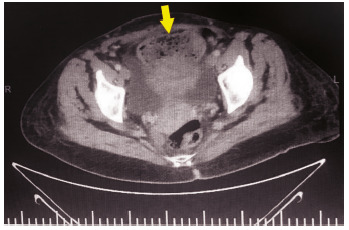
Sagital computerized tomography view (yellow arrow=gossybipoma)

Gossypiboma was removed from five cases (Figures 1 and 2). The mean age was 42±10.27 (27-54) years and the ratio of female/male was 1/4. The longest diagnosis period after the first operation was two years and the shortest was the third day after surgery (Table 1). 

**TABLE 1:** Demographic values of the patients

Patient	Age	Gender	Previous disease	Performed surgery	Time passed after first surgery	Compliant	Diagnostic method	The second surgery
1	27	M	Acute appendicitis.	Appendectomy	4 days	Abdominal pain, nausea, vomiting	Laparoscopic exploration	Laparoscopic foreign body excision
2	38	F	Ileus after C/S	C/S	5 days	Abdominal pain, nausea, vomiting	Oral + IV contrast-abdominal CT	Foreign body excision
3	43	F	Uterine atony after C/S	Hysterectomy	3 days	Ileus	IV contrast- abdominal CT	Foreign body excision
4	54	F	--	Hysterectomy	1 year	Abdominal distension, indigestion, abdominal pain	Oral + IV contrast-abdominal CT	Small intestine resection + foreign body excision
5	48	F	Right breast CA	Right MRM	2 years	Swelling at the site of the wound	U/S PET-CT	Foreign body excision

## DISCUSSION

The incidence of gossypiboma has been reported less frequently not because of the legal consequences of its findings, but also because many patients remain asymptomatic. However, if a number is to be given, 1/1000 to 1/1500 occur in intra-abdominal operations. Clinical presentation is variable and depends on the location of the foreign body and the type of body reaction^4^ ^,^ ^5^. Forgotten intraabdominal foreign bodies manifest themselves with ileus, intra-abdominal masses, postoperative abdominal pain, nausea and vomiting. Again, extra-abdominal forgotten objects should be differentiated from malignancies due to mass like image.

Imaging procedures for diagnosis are mainly aimed at revealing the cause. Ultrasonography is a cheap, easy to use diagnostic method in every hospital for intra-abdominal etiological explorations. It may also help in the differential diagnosis of extra-abdominal lesions as in our 5^th^ case. However, it may not be sufficient to evaluate the abdominal organs in cases such as ileus. Computed tomographic examinations will be useful for the differential diagnosis of ileus findings. In this way, an obstructive lesion or adhesions due to previous operations can be distinguished.

Once the diagnosis has been made, it should be removed even if the patient is asymptomatic. Open or laparoscopic technique may be chosen in preliminary diagnosis of gossypiboma^6^. We performed laparoscopy in the patient who underwent open appendectomy and re-exploration due to abdominal pain. We performed re-laparotomy in other patients. The most important advantage of laparoscopy is of course whole abdominal exploration. Laparoscopy should be planned and applied especially in suspicious cases. While surgical excision of gossypibomas in the extra-abdominal area is performed, previous diagnosis should be taken into consideration. As in our 5^th^ patient with a history of malignancy surgery, the excision of these structures should still be appropriate for cancer surgery.

The primary requirement to diagnose is suspicion. However, most of the time, it can be diagnosed as a result of tomography. Their characteristic appearance is that they have a spongy appearance^7^. However, in some studies, it is emphasized that differential diagnosis should be made especially with fungal infections^2^ ^,^ ^8^. Also in this study, tomography was used to detect foreign bodies that were forgotten especially in the abdomen. Axillary involvement is evaluated as recurrence and lymphadenopathy after PET-CT imaging during oncologic follow-up for the body in the axilla. Ultrasonography had a pre-diagnosis of foreign body.

When the literature is examined, it is determined that gossypibomas are mostly reported after abdominal and pelvic surgeries^1^. However, it has also been reported after thoracic surgery, spinal, orthopedic and breast surgery^2^ ^,^ ^3^. Our study, which has a small number of cases, reviewed the situation in the areas related to the general surgery clinic and is naturally mostly from the abdomen. In one of our cases, forgotten sponge after a modified radical mastectomy was removed from the axilla, similar to Boussaid et al.^2^.

The incidence of gossypiboma is increasing and, more importantly, it is a legal problem^5^. For this reason, preventive measures should be taken in the operating room, and especially the scrub nurses and assisted health personnel should be trained on the subject. 
